# Impact of engineering renovation on dynamic health risk assessment of mercury in a thermometer enterprise

**DOI:** 10.3389/fpubh.2022.1037915

**Published:** 2022-11-14

**Authors:** Peihong Wu, Jianrui Dou, Yanqiong Xu, Zhengmin Yu, Lei Han, Baoli Zhu, Xin Liu, Hengdong Zhang

**Affiliations:** ^1^Department for the Prevention and Treatment of Occupational Diseases, Institute of Occupational Disease Prevention, Jiangsu Provincial Center for Disease Control and Prevention, Nanjing, China; ^2^Jiangsu Province Engineering Research Center of Health Emergency, Nanjing, China; ^3^Jiangsu Preventive Medicine Association, Nanjing, China; ^4^Scenic Area Division, Yangzhou Center for Disease Control and Prevention, Yangzhou, China

**Keywords:** mercury, thermometer, occupational health risk assessment, renovation, risk ratio, concentration, occupational exposure

## Abstract

The occupational health risk assessments (OHRA) of inorganic mercury (Hg) are rarely reported. We conducted an internal and external exposure monitoring of employees in a thermometer enterprise which experienced the renovation of occupational health engineering, followed by an evaluation on the health risks of Hg exposure with four OHRA methods in order to find out a most suitable model. The results showed that the concentrations of airborne and urinary Hg in all testing positions and subjects obviously decreased after the engineering renovation, meeting the occupational exposure limits (OELs) of China. Subsequently, four OHRA models, namely the models from US Environmental Protection Agency (EPA), Ministry of Manpower (MOM), International Council on Mining and Metals (ICMM), and Classification of occupational hazards at workplaces Part 2: Occupational exposure to chemicals (GBZ/T 229.2-2010) were applied in the qualitative risk assessment. And the evaluation results of different methods were standardized by risk ratio (*RR*), which indicated MOM, ICMM risk rating, and GBZ/T 229.2 models were consistent with the order of inherent risk levels in those working processes. The order of *RR* between four models was: *RR*_EPA_ > *RR*_ICMM_ > *RR*_MOM_> *RR*_GBZ/T229.2_ (*P* < 0.05). Based on the strict limits of Hg, GBZ/T 229.2, and MOM methods may have more potentials in practical application. Though the working environment has been significantly improved via engineering renovation, it is strongly suggested that the thermometer company conduct more effective risk management covering all production processes to minimize Hg exposure levels and health risk ratings.

## Introduction

Mercury-containing thermometers are widely used in medical institutions because of their stable performance, convenient operation, and low price (1). Mercury, as the only liquid metal element on the earth (2), is recognized by the World Health Organization (WHO) as one of the top 10 chemicals or groups of chemicals of major public health concern (3), which is the most common chemical hazardous agent for thermometer manufacturing enterprises. It often invades the human body in the form of vapor during production activities, and long-term exposure can cause occupational mercury poisoning, affecting the nervous system, the digestive system, and the immune system, and damaging human health. In recent years, domestic and foreign scholars have identified and analyzed workplace hazards through occupational health risk assessments (OHRA) (4–8), and many researchers have conducted corresponding studies on the occupational hazard risks of mercury (9, 10). Zhu et al. (11) studied the characteristics of mercury pollution at the site of a thermometer manufacturer and conducted the health risk assessment. Han et al. (12) used the Environmental Protection Agency (EPA) model to assess the non-carcinogenic risk of mercury in fluorescent lamp manufacturing enterprises, and found that the mercury concentration in the exhaust mercury-injecting post exceeded the standard, which was high risk. Ruan et al. (13) used the Ministry of Manpower (MOM) model to assess occupational hazards in energy-saving lamp production enterprises, and found this model can objectively reflect the actual risk level of the workplace. In this paper, EPA, MOM, International Council on Mining and Metals (ICMM), and GBZ/T 229.2 (14) were used to carry out OHRA of mercury in a thermometer enterprise, comparing the risk differences before and after the renovation of occupational disease protection facilities longitudinally, and focusing on the correlation between risk levels and occupational exposure under different methods transversely, to obtain suitable methods for dynamic occupational risk assessment of mercury.

## Object and methods

### Object

**Figure 1:**
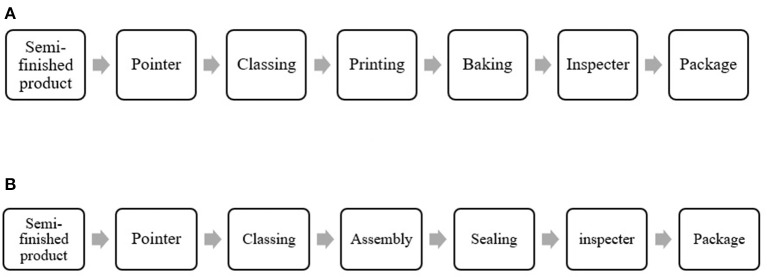
Main technological process of the thermometer manufacturing enterprise: **(A)** Trigonal thermometer, **(B)** Internal scaling thermometer.

A thermometer manufacturing enterprise in Jiangsu was selected to conduct on-site testing and analysis in December 2019 and September 2020. The products of the enterprise conclude trigonal thermometers and internal scaling thermometers. The technological process can be seen in Figure 1. According to the early survey, the main occupational health hazard is mercury. The posts where workers could be exposed to mercury were all taken into considerations.

### Methods

Five OHRA models were used to classify the risk of occupational diseases for employees, including EPA, MOM, ICMM, and GBZ/T 229.2. Several representative posts were chosen to carry out short time sampling in accordance with GBZ/T 159-2004: Sampling Practices for Monitoring Harmful Substances in workplace Air (15). The detection factor was tested according to GBZ/T 300.18-2017: determination of toxic substances in workplace air-Part 18: Mercury and its compound (16). The occupational exposure limits (OELs) in China stipulate that the 8-h time-weighted average allowable concentration of mercury is 0.02 mg/m^3^. The judgment of whether the concentration of mercury in the workplace exceeds the standard is made according to the OELs.

#### EPA

In this model, the method can be divided into cancer risk assessment and non-carcinogenic risk assessment, having two steps in the process of OHRA: exposure concentration (*EC*) estimation and health risk assessment (17–19). *EC* is determined by *CA, ET, EF, ED*, and *AT*, as calculated by Equation (1):


(1) $$\begin{array}{l} EC=\left(CA\;*\;ET\;*\;EF\;*\;ED\right)/AT \end{array}$$

where *CA* is the concentration of the toxic and hazardous chemicals in the air of the workplace (g/m^3^). *ET* is the exposure time of employees in the workplace (h/day). *EF* is the exposure frequency of employees in the workplace (day/year). *ED* is the duration of exposure during the exposure period (y). *AT* is the average exposure time (h), the value of which is ED^*^24^*^365. The non-carcinogenic risk, hazard quotient (*HQ*) of mercury is calculated by Equation (2):


(2) $$\begin{array}{l} HQ=EC/RfC \end{array}$$

where *RfC* is the inhalation toxicity reference value of the toxicant to be evaluated, also known as the reference concentration (mg/m^3^). The *RfC* of mercury is 0.3 μg/m^3^ according to the IRIS database.

#### MOM

In the MOM semiquantitative risk assessment model (20, 21), the risk is determined by hazard level (*HR*) and exposure level (*ER*). The hazard classification of chemicals is divided by toxicity of the chemicals with five levels: no risk (grade 1); low risk (grade 2); moderate risk (grade 3); high risk (grade 4); extreme risk (grade 5), and that of mercury is 5 (13, 22). *ER* is determined by comparing the weekly time-weighted average exposure level (*E*) with the long-term OEL. *E* is calculated by Equation (3) (23):


(3) $$\begin{array}{l} E=\left(F\;*\;D\;*\;M\right)/W \end{array}$$

where *F* is the weekly exposure frequency; *D* is the average exposure time (h); *M* is the air detecting concentration (PPM or mg/m^3^); *W* is the average working time per week (40 h). The risk is calculated by Equation (4):


(4) $$\begin{array}{l} R=\sqrt{HR\;*\;ER} \end{array}$$

#### ICMM

The ICMM model involves two methods (24, 25), one is ICMM risk rating method, and the other is ICMM quantitative method of assignment. The former determines the risk level based on the level of occupational exposure, the effectiveness of protection, and the likelihood of occupational exposure, depending on subjective judgment to a great extent. When using ICMM quantitative method of assignment, the occupational risk is calculated by Equation (5):


(5) $$\begin{array}{l} rr=C\;*\;PrE\;*\;PeE\;*\;U \end{array}$$

When *rr* is risk rank, *C* is the occupational health consequences, according to the degree of harm, the value of mercury in this study is 100. *PrE* is exposure probability, which is assigned according to the result of onsite testing: <50% OEL is assignment 3; 50–100% OEL is assignment 6; ≥100% OEL is assignment 10. *PeE* is exposure time. Supplemental Table 1 presents the assignment. *U* is the uncertainty parameter: certainty is assignment 1; uncertain is assignment 2; very uncertain is assignment 3. Risk grades are determined by *rr*, as shown in Supplemental Table 2.

#### GBZ/T 229.2

GBZ/T 229.2 considers the hazard of chemicals, occupational exposure ratio, and physical workload of workers. The weights of the three factors correspond to *W*_*D*_, *W*_*B*_, and *W*_*L*_, respectively, and the values are shown in Supplemental Tables 3–5. *W*_*B*_ is determined by *B*, which is the ratio of occupational exposure level to OELs in particular.

The grading index of occupational hazards is defined as *G*, calculated by Equation (6), corresponding to four types of operations, as illustrated in Supplemental Table 6.


(6) $$\begin{array}{l} G=W_{D}\;*\;W_{B}\;*\;W_{L} \end{array}$$

#### Standardization of assessment results

To better compare the assessment results of different models, the risk ratio (RR) was put forward to standardize the occupational risk, which was calculated by Equation (7),


(7) $$\begin{array}{l} RR=\frac{Risk\;Grade\;}{Total\;Grade} \end{array}$$

In the method of EPA, MOM, and ICMM quantitative method of assignment, there are five risk levels corresponding to the risk grades 1–5, and the larger the value, the higher the risk level. While in the method of ICMM risk rating and GBZ/T 229.2, the total grade is 4.

### Statistics

SPSS 27.0 software was used for statistical analysis. *RR* between the 2 years were tested by a non-parametric test. *P* < 0.05 was considered statistically significant. Kendall's *W*-test was executed to assess agreement among the *RRs* obtained from different OHRA models, which was a non-parametric statistic. Kendall's coefficient of concordance *W* ranges from 0 (no agreement) to 1 (complete agreement) (26, 27). Spearman correlation analysis was used to analyze the correlation between *RRs* and occupational exposure, and *P* < 0.05 was considered significant.

## Results

### Results of on-site survey and mercury concentration tests

As illustrated in Table 1, the concentrations of mercury in 75% of the posts were beyond the OEL in 2019. In terms of the on-site survey, the main control measures applied throughout the factory include isolating equipment, submerging broken thermometers in trays of water, and conducting a continuous clean-up program. Actually, the size of the isolation cabinet did not fit well with the degassing machine, leaving doors not fully closed. The floor of the rooms was laid by terrazzo, and the surface of the walls was uneven. The height of the side wall exhaust fans was set too high. There was no exhaust hood installing at the mouth of the crusher. The exhaust gas treatment device was set on the top of the workshops, greatly affecting the efficiency of ventilation and detoxification. What's more, the number of tail gas treating units was small. In a word, the lack of rationality in the setting of protective measures for occupational diseases was the main reason for the excessive mercury concentration.

**Table 1:** Results of mercury concentration tests in 2019 (mg/m^3^).

**Production process**	**Post**	**Duration of exposure**	**Median (Range)**	**TWA**	**STEL**	**Judgment**
Trigonal thermometer	Pointer	10	0.073 (0.071–0.074)	0.091	0.074	Unqualified
	Classing	10	0.061 (0.025–0.074)	0.066	0.074	Unqualified
	Printing	10	0.053 (0.035–0.063)	0.063	0.063	Unqualified
	Baking	10	0.039 (0.037–0.040)	0.039	0.040	Unqualified
	Package	10	0.050 (0.023–0.070)	0.060	0.070	Unqualified
Internal scaling thermometer	Pointer	10	0.018 (0.013–0.028)	0.024	0.028	Unqualified
	Classing	10	0.013 (0.011–0.020)	0.019	0.020	Qualified
	Assembly	8	0.018 (0.011–0.019)	0.016	0.019	Qualified
	Sealing	8	0.024 (0.013–0.024)	0.020	0.024	Qualified
	Package	10	0.023 (0.017–0.028)	0.029	0.028	Unqualified
Inspection area	Inspecter	10	0.023 (0.014–0.029)	0.028	0.029	Unqualified
Solid waste treatment	Crushing	0.8	0.115 (0.105–0.254)	0.016	0.254	Unqualified

In 2020, the enterprise experienced the renovation of occupational health engineering, and the main measures were as followed, laying smooth pads on the workbench, setting up a local exhaust hood at the workstation, changing the location of the exhaust gas treatment device from the high position to the low position, etc. These measures greatly promoted the emissions of inorganic mercury vapor. All the operations involving mercury were performed over impermeable surfaces without crevices, which helped to reduce the mercury exposure of workers. In a word, the engineering facilities appeared to run in good condition compared to that in 2019. In terms of process transformation, the fixed point and packaging process of the two thermometers were merged. The test results shown in Table 2 indicated that the mercury concentration of each post after the transformation was qualified. And the mean level of TWA in 2020 decreased significantly (*P* = 0.002) in comparison with that of 2019, indicating that engineering renovation greatly reduced the mercury concentration in the air of workplaces. Furthermore, the internal exposures of the subjects were analyzed. The urinary Hg values declined obviously among 51 frontline workers after engineering renovation (median levels: 132.1 μg/g Cr before the renovation and 54.9 μg/g Cr after the renovation, *P* < 0.001).

**Table 2:** Results of mercury concentration tests in 2020 (mg/m^3^).

**Production process**	**Post**	**Duration of exposure**	**Median (Range)**	**TWA**	**STEL**	**Judgment**
Trigonal thermometer	Classing	8	0.009 (0.003–0.018)	0.016	0.018	Qualified
	Printing	8	0.010 (0.006–0.019)	0.015	0.019	Qualified
	Baking	8	0.011 (0.003–0.015)	0.013	0.015	Qualified
Internal scaling thermometer	Classing	8	0.004 (0.002–0.008)	0.006	0.008	Qualified
	Assembly	8	0.005 (0.002–0.011)	0.007	0.011	Qualified
	Sealing	8	0.008 (0.005–0.017)	0.012	0.017	Qualified
	Pointer	8	0.010 (0.005–0.018)	0.011	0.018	Qualified
	Package	8	0.003 (0.002–0.006)	0.004	0.006	Qualified
Inspection area	Inspecter	8	0.010 (0.004–0.017)	0.013	0.017	Qualified
Solid waste treatment	Crushing	4	0.010 (0.004–0.015)	0.007	0.015	Qualified

### Results of OHRA

The risk assessment results of each model were shown in Tables 3, 4. Table 5 illustrated the percentage of posts with different risk. In 2019, the assessment results of EPA model and ICMM quantitative method of assignment were consistent. Over 90% of the posts were unacceptable risks, corresponding to the risk rating scaling of 5. As a result of MOM, 33% of the posts were extremely high risk, mainly distributed in the triangular thermometer production area. The risk of the other posts was high. In all, the general risk level of MOM model is lower than that of EPA model and ICMM quantitative method of assignment. When using ICMM risk rating method, 75% of the posts were extremely high risk. The results of GBZ/T 229.2 showed that 42% of the posts were severe hazard operations, and 33% of the posts were moderate hazard operations.

**Table 3:** Results of the health risk assessment of mercury exposure at each position in 2019.

**Post**	**EPA**			**MOM**			**ICMM risk rating method**				**ICMM quantitative method of assignment**					**GBZ/T 229.2**				
	***EC* (μg/m^3^)**	***HQ***	**Risk level**	**E/PEL**	***ER***	**Risk level**	**Exposure level**	**Effectiveness of protective facilities**	**Likelihood of exposure occurring**	**Risk level**	***PrE***	***PeE***	***U***	**Risk value**	**Risk level**	***W_*D*_***	***W_*B*_***	***W_*L*_***	***G***	**Classification**
Pointer-T	27.01	90.03	5	4.55	5	5	High	Poor	Medium	4	6	10	1	6,000	5	8	4.55	1.5	55	III
Classing-T	19.59	65.30	5	3.30	5	5	High	Poor	Medium	4	6	10	1	6,000	5	8	3.3	1.5	40	III
Printing-T	18.70	62.33	5	3.15	5	5	High	Poor	Medium	4	6	10	1	6,000	5	8	3.15	1.5	38	III
Baking-T	11.58	38.58	5	1.95	4	4	High	Poor	Medium	4	6	10	1	6,000	5	8	1.95	1.5	23	II
Package-T	17.81	59.36	5	3.00	5	5	High	Poor	Medium	4	6	10	1	6,000	5	8	3	1.5	36	III
Pointer-I	7.12	23.74	5	1.20	4	4	High	Poor	Medium	4	6	10	1	6,000	5	8	1.2	1.5	14	II
classing-I	5.64	18.80	5	0.95	3	4	Medium	Poor	Medium	3	6	10	1	6,000	5	8	0	1.5	0	0
Assembly-I	3.80	12.66	5	0.80	3	4	Medium	Poor	Medium	3	6	10	1	6,000	5	8	0	1.5	0	0
Sealing-I	4.75	15.83	5	1.00	4	4	High	Poor	High	4	6	10	1	6,000	5	8	1	1.5	12	II
Package-I	8.61	28.69	5	1.45	4	4	High	Poor	Medium	4	6	10	1	6,000	5	8	1.45	1.5	17	II
Inspecter	8.31	27.70	5	1.40	4	4	High	Poor	High	4	6	10	1	6,000	5	8	1.4	2	17	II
Crushing	0.38	1.27	4	0.80	3	4	Medium	Poor	High	3	6	2	1	1,200	5	8	0	2	0	0

**Table 4:** Results of the health risk assessment of mercury exposure at each position in 2020.

**Post**	**EPA**			**MOM**			**ICMM risk rating method**				**ICMM quantitative method of assignment**					**GBZ/T 229.2**				
	**EC (μg/m^3^)**	***HQ***	**Risk Level**	**E/PEL**	***ER***	**Risk Level**	**Exposure level**	**Effectiveness of protective facilities**	**Likelihood of exposure occurring**	**Risk level**	***PrE***	***PeE***	***U***	**Risk value**	**Risk level**	***W_*D*_***	***W_*B*_***	***W_*L*_***	***G***	**Classification**
Classing-T	3.80	12.66	5	0.80	3	4	Medium	Good	Medium	3	6	10	1	6,000	5	8	0	1.5	0	0
Printing-T	3.56	11.87	5	0.75	3	4	Medium	Good	Medium	3	6	10	1	6,000	5	8	0	1.5	0	0
Baking-T	3.09	10.29	5	0.65	3	4	Medium	Good	Medium	3	6	10	1	6,000	5	8	0	1.5	0	0
Classing-I	1.42	4.75	5	0.30	2	3	Rare	Good	Medium	2	6	10	1	6,000	5	8	0	1.5	0	0
Assembly-I	1.66	5.54	5	0.35	2	3	Rare	Good	Medium	2	6	10	1	6,000	5	8	0	1.5	0	0
Sealing-I	2.85	9.50	5	0.60	3	4	Medium	Good	High	3	6	10	1	6,000	5	8	0	1.5	0	0
Pointer	2.61	8.71	5	0.55	3	4	Medium	Good	Medium	3	6	10	1	6,000	5	8	0	1.5	0	0
Package	0.95	3.17	5	0.20	2	3	Rare	Good	Medium	2	6	10	1	6,000	5	8	0	1.5	0	0
Inspecter	3.09	10.29	5	0.65	3	4	Medium	Good	High	4	6	10	1	6,000	5	8	0	1.5	0	0
Crushing	0.83	2.77	5	0.35	2	3	Rare	Good	High	3	6	6	1	3,600	5	8	0	1.5	0	0

**Table 5:** Post risk distribution in 2019 and 2020.

**Method**	**Percentage of posts with extremely high risk in 2019**	**Percentage of posts with high risk in 2019**	**Percentage of posts with extremely high risk in 2020**	**Percentage of posts with high risk in 2020**
EPA	92%	8%	100%	0
MOM	33%	67%	0	60%
ICMM risk rating method	75%	25%	10%	60%
ICMM quantitative method of assignment	100%	0	100%	0
GBZ/T 229.2	42%	33%	0	0

In 2020, the results of MOM and ICMM quantitative method of assignment remained unchanged, maintaining the level of extremely high risk. In MOM and ICMM risk rating methods, the risk level of major posts declined significantly, and the number of high-risk posts decreased. The posts of pointer, classing, printing, and package in the triangular thermometer production area changed from extremely high risk to high risk. In the method of GBZ/T 229.2, there was no post with severe hazard operation in 2020. All the posts were relatively harmless operations.

Tables 6, 7 show *RRs* of different models. The order of *RRs* between four models was: *RR*_EPA_ > *RR*_ICMM_ > *RR*_MOM_ > *RR*_GBZ/T229.2_ (*P* < 0.05) on the whole. There was no significant difference in the risk level before and after the transformation using EPA and ICMM assignment quantitative methods. Among the three assessment methods of MOM, ICMM risk rating method, and GBZ/T 229.2, *RRs* in 2020 were significantly reduced compared with 2019. Non-parametric tests were used to analyze the differences of *RRs* for each assessment model in 2019 and 2020. The results showed that the risk level of each post changed significantly after the transformation of occupational disease protection facilities in 2020 (MOM model, *P* = 0.006; ICMM risk rating method, *P* = 0.002; GBZ/T 229.2, *P* = 0.002).

**Table 6:** *RRs* of exposure to mercury at each position in 2019.

**Post**	**EPA**	**MOM**	**ICMM**		**GBZ/T 229.2**
			**Risk rating method**	**Quantitative method of assignment**	
Pointer-T	1	1	1	1	1
Classing-T	1	1	1	1	1
Printing-T	1	1	1	1	1
Baking-T	1	0.8	1	1	0.75
Package-T	1	1	1	1	1
Pointer-I	1	0.8	1	1	0.75
classing-I	1	0.8	0.75	1	0.25
Assembly-I	1	0.8	0.75	1	0.25
Sealing-I	1	0.8	1	1	0.75
Package-I	1	0.8	1	1	0.75
Inspecter	1	0.8	1	1	0.75
Crushing	0.8	0.8	0.75	1	0.25

**Table 7:** *RRs* of exposure to mercury at each position in 2020.

**Post**	**EPA**	**MOM**	**ICMM**		**GBZ/T 229.2**
			**Risk rating method**	**Quantitative method of assignment**	
Classing-T	1	0.8	0.75	1	0.25
Printing-T	1	0.8	0.75	1	0.25
0.75 ng-T	1	0.8	0.75	1	0.25
Classing-I	1	0.6	0.5	1	0.25
Assembly-I	1	0.6	0.5	1	0.25
Sealing-I	1	0.8	0.75	1	0.25
Pointer	1	0.8	0.75	1	0.25
Package	1	0.6	0.5	1	0.25
Inspecter	1	0.8	1	1	0.25
Crushing	1	0.6	0.75	1	0.25

The results of Kendall's coefficient of concordance *W*-test illustrated that the *RRs* obtained from the model of MOM, ICMM risk rating method, and GBZ/T 229.2 were comparable (2019, *W* = 0.51, *P* < 0.05; 2020, *W* = 0.8, *P* < 0.05). To further compare the applicability of MOM, ICMM risk rating method, and GBZ/T 229.2, the correlation analysis between *RR* and TWA was carried out, as shown in Table 8. Significant correlation was found in this study, indicating that the three models apply to the OHRA of mercury, among which the applicability of GBZ/T 229.2 is the best, followed by the MOM model, and finally ICMM risk rating method.

**Table 8:** Results of Spearman correlation analysis.

**Method**	***r_*s*_***	***P***
MOM	0.821	0.001
ICMM risk rating method	0.754	0.005
GBZ/T 229.2	0.94	<0.001

## Discussion

In the comparison of results of on-site surveys in 2019 and 2020, the main changes happened in the renovation of protective facilities, which directly affects the concentration of mercury in the air of workplaces. There were few changes in the use of personal protective equipment and the occupational health management over the 2 years. The analysis was carried out from two dimensions.

From the vertical perspective, the most intuitive change in this dynamic assessment was a significant reduction in on-site mercury concentration. However, from the assessment results of EPA model and ICMM quantitative method of assignment, there was no statistically significant difference in the *RRs* over the 2 years. Environmental Protection Agency model is a comprehensive and quantitative method, and there are several factors involved in the OHRA of mercury, including the concentrations, exposure time, exposure frequency, and working ages, which is suitable for assessing the long-term chronic effects of substances. On one hand, in the comparison of assessment factors of the 2 years, factors except the concentrations remained unchanged, and the concentration had weak influence on the assessment results. On the other hand, The *RfC* used in EPA model is 0.3 μg/m^3^, having a smaller order of magnitude compared to exposure concentration. Under the premise that the mercury concentration decreased significantly, the calculated *HQ* was still large, so the risk level did not change significantly, which reflected the limitations of the model in dynamic assessment during a short period of time with changes in the mercury concentration in the workplaces. What's more, the calculation of the model is based on the IUR and *RfC* of chemicals in the IRIS database in the United States, which cannot be used to assess the occupational health risk of chemicals that are not included in the database.

In the model of ICMM quantitative method of assignment, which was refined based on the ICCM risk rating method, the factor of exposure time was taken into consideration. Similar to the EPA model, the change of the single factor of concentration in a short period of time did not have significant impacts on the evaluation results. What's more, the assignment range of the four parameters varies greatly, which can easily amplify the risk level and reflect the high requirements of occupational health protections in the mining industry. When the assignment of material consequences is large, the risk value can easily exceed the threshold, and the overall assessment result is high. It is recommended to adjust the assignment range of the four parameters to refine the division of risk levels when the method was used in other industries. Although the risk level was judged by the specific values, the model was still considered as a qualitative assessment method. At the same time, there is a need for evaluators to extensively review and discuss data to reduce subjective bias.

From the horizontal perspective, *RRs* derived from the three methods applicable to dynamic risk assessment of mercury also differed in their association with occupational exposure. International Council on Mining and Metals risk rating method is based on the actual exposure concentration of the substance, but the effectiveness of the protective facilities and the possibility of exposure depend on subjective judgment. The evaluation parameters are few and the operability is strong, but the stability of the evaluation results needs to be strengthened. The semi-quantitative characteristics of the MOM model can objectively reflect the risk level of the evaluation system. In the calculation process, the exposure level is assigned according to the exposure concentration, which has been widely used in the OHRA of chemical substances. However, it cannot be used for risk assessment of physical occupational hazards such as high temperature and noise. The method of GBZ 229.2 was the most practical in this study, in which the assessment process considered the degree of harm of chemical substances, occupational exposure, and the intensity of manual labor of workers. It was also improved since the *RRs* were obtained after standardizing the grading results regarding foreign methods. Compared with MOM's assignment of exposure level, this method directly used the on-site detection concentration to calculate the classification index, so the *RRs* and occupational exposure correlation in GBZ/T 229.2 are the most significant.

## Conclusions

Mercury is an ancient and traditional poison. However, there are still few studies on OHRA of mercury in thermometer manufacturers. In this study, multiple methods were used to carry out the OHRA of mercury. The results showed that the mercury exposure was significantly improved after the renovation of occupational protection facilities, while the EPA and ICMM quantitative method of assignment failed to reflect this change and may not be suitable for the dynamic assessment of occupational health risks of mercury. The model of MOM, ICMM risk rating method, and GBZ/T 229.2 have good applicability in this study, the applicability of GBZ/T 229.2 is the best, followed by MOM, and finally the ICMM risk rating method. What's more, though the working environment has been significantly improved via engineering renovation, it is strongly suggested that the thermometer enterprise conduct more effective risk management covering all production processes to minimize Hg exposure levels and health risk ratings.

This study focused on the occupational health risks of mercury in different years. Sustained attention can be paid to the concentration of mercury exposure in major positions to obtain more data, which can be used to monitor job risk and optimize the existing risk assessment model.

## Data availability statement

The original contributions presented in the study are included in the article/Supplementary material, further inquiries can be directed to the corresponding author/s.

## Ethics statement

The studies involving human participants were reviewed and approved by the Ethical Committee of Jiangsu Provincial Center for Disease Control and Prevention (REF JSJK2022-B002-01). The participants provided their written informed consent to participate in this study.

## Author contributions

PW and JD participated in the analysis of the data and interpretation of the results and wrote the first draft of the manuscript. XL, HZ, and BZ contributed to the conception and design of the study. YX, LH, ZY, and HZ were involved in the fieldwork, sample collection, and processing. All authors contributed to manuscript revision, read, and approved the submitted version.

## Funding

This work was funded by Key Research and Development Program of Jiangsu Commission of Health (ZD2021024), Jiangsu Province's Outstanding Medical Academic Leader Program (CXTDA2017029), and Yangzhou Science and Technology Development Plan Project (YZ2022084).

## Conflict of interest

The authors declare that the research was conducted in the absence of any commercial or financial relationships that could be construed as a potential conflict of interest.

## Publisher's note

All claims expressed in this article are solely those of the authors and do not necessarily represent those of their affiliated organizations, or those of the publisher, the editors and the reviewers. Any product that may be evaluated in this article, or claim that may be made by its manufacturer, is not guaranteed or endorsed by the publisher.

## Supplementary material

Supplemental Tables 1–6 Illustrated the values of the main indexes used in the several OHRA models.
