# Supplementary material for: Impact of engineering renovation on dynamic health risk assessment of mercury in a thermometer enterprise
Source: Front Public Health. 2022 Nov 14;10:1037915. doi: 10.3389/fpubh.2022.1037915 (PMC9704025; doi:10.3389/fpubh.2022.1037915)
Supplement: Supplemental Tables 1–6 — Illustrated the values of the main indexes used in the several OHRA models. [file Table_1.DOCX]

# Supplemental Table 1 Assignment of *PeE*

| Exposure time | Assignment |
| --- | --- |
| Once a year | 0.5 |
| Several times a year | 1 |
| Several times a month | 2 |
| Continuous exposure for 2 to 4 h per shift | 6 |
| Continuous exposure for 8 h per shift | 10 |

# Supplemental Table 2 Risk level according to *rr*

| *rr* | Assignment |
| --- | --- |
| ≥400 | unacceptable risk |
| 200~399 | very high risk |
| 70~199 | high risk |
| 20~69 | potential risk |
| <20 | tolerable risk |

# Supplemental Table 3 Value of *W_D_*

| Hazard | *W_D_* |
| --- | --- |
| Mild hazard | 1 |
| Moderate hazard | 2 |
| Severe hazard | 3 |
| Extreme hazard | 4 |

# Supplemental Table 4 Value of *W_B_*

| *B* | *W_B_* |
| --- | --- |
| *B*≤1 | 0 |
| *B*>1 | *B* |

# Supplemental Table 5 Value of *W_L_*

| Physical workload of workers | *W_L_* |
| --- | --- |
| Mild | 1.0 |
| Moderate | 1.5 |
| Severe | 2.0 |
| Extreme | 2.5 |

# Supplemental Table 6 Classification of occupational hazards.

| *G* | Classification |
| --- | --- |
| ≤1 | 0 (relatively harmless operation) |
| 1＜*G*≤6 | Ⅰ (mildly hazardous operation) |
| 6＜*G*≤24 | Ⅱ (moderately hazardous operation) |
| >24 | Ⅲ (Severely hazardous operation) |
